# Vitamin D regulation of immune function during covid-19

**DOI:** 10.1007/s11154-021-09707-4

**Published:** 2022-01-29

**Authors:** Daniel D. Bikle

**Affiliations:** Veterans Affairs Medical Center and University of California San Francisco, 4150 Clement Street (111N), San Francisco, CA 94121 USA

**Keywords:** Vitamin D, Calcitriol, Innate immunity, Adaptive immunity, Pulmonary alveolar macrophage, Airway epithelia, Viral infection, Cathelicidin

## Abstract

Covid-19 has to date infected a confirmed 275 million people with 5.4 million, now dead, with the count rising every day. Although the virus, SARS-CoV2, causing Covid-19 infects many cells in the body, its infection of the upper and lower respiratory tract (upper airway epithelia and pulmonary alveolar pneumocytes and macrophages) causing what is now called a cytokine storm in the lungs is the major cause of morbidity and mortality. This results from a dysregulation of the innate immune system with an outpouring of proinflammatory cytokines and chemokines leading to abnormal activation of the adaptive immune pathway. Airway epithelia constitutively expresses CYP27B1, the enzyme producing the active vitamin D metabolite, 1,25(OH)_2_D, and the vitamin D receptor (VDR) for which 1,25(OH)_2_D is the ligand. Pulmonary alveolar macrophages, on the other hand, are induced to express both CYP27B1 and VDR by various pathogens including viruses and cytokines released from infected epithelia and other immune cells. Although not demonstrated for corona viruses like SARS-CoV2, for other viruses and other respiratory pathogens activation of innate immunity leading to increased local 1,25(OH)_2_D production has been shown to enhance viral neutralization and clearance while modulating the subsequent proinflammatory response. Whether such will be the case for SARS-CoV2 remains to be seen, but is currently being proposed and investigated. This mini review will discuss some of the mechanisms by which vitamin D may help reduce morbidity and mortality in this devastating pandemic.

## Introduction

At the time of writing (June 1, 2021) SARS-CoV2, the virus causing the Covid-19 pandemic has infected nearly 275 million people causing approximately 5.4 million deaths world wide, with more each day. Because testing and/or reporting is spotty in a number of countries, the likely count is much higher. The clinical and immunologic features of Covid-19 are only recently being examined given its recent appearance [1], but the findings bear close resemblance to the SARS-CoV1 epidemic from 2002–2003, about which more is known. SARS-CoV1 and 2 share approximately 80% homology [2]. Therefore, it is useful to review the immunologic consequences of the better studied SARs-CoV1 infection, primarily from animal studies, as a background for examining how vitamin D signaling might play a role in the current epidemic. That said Chu et al. [3] noted in bronchial pulmonary lavage material from patients with either SARS-CoV1 or SARS-CoV2 that the SARS-CoV2 virus infected and replicated faster in alveolar pneumocytes and macrophages than did SARS-CoV1 but did not induce interferons and other proinflammatory cytokines as well, perhaps accounting for its overall greater infectivity but lower mortality.

The SARS-CoV2 virus enters the cell via its spike protein, which has two functional subunits. The S1 subunit binds the receptor, the enzyme ACE2 (angiotensin converting enzyme 2). The other subunit, S2, promotes the fusion of the viral and host membrane leading to its internalization. Epithelial cells of the upper airway have the highest concentration of ACE2, but those of the lower airway (alveolar pneumocytes) do as well [4, 5]. Thus, if the infection is confined to the upper airways the subject may remain asymptomatic although infectious. Moreover, the lower expression of ACE2 in the nasal epithelium of younger children has been postulated as a reason for their decreased infection rate compared to older children and adults [6, 7]. In addition to ACE2, the serine protease TMPRSS2 also appears necessary to cleave the SARS-CoV2 protein enabling its membrane fusion and endocytosis. Co-expression of both ACE2 and TMPRSS2 is found in only a small percentage of epithelial cells in both the upper and lower respiratory tract [4, 8]. However, ACE2 also serves a protective role. It cleaves angiotensin II to angiotensin 1–7, limiting renin angiotensin system (RAS), which can suppress inflammation and fibrosis while increasing vasodilatation by binding to the MAS receptor. Mice lacking ACE2 undergo severe lung injury [9]. SARS-CoV2 infection reduces ACE2 levels [10], possibly contributing to its inflammatory capacity. On the other hand vitamin D increases ACE2 expression while inhibiting the RAS system [11] thought to contribute to its role in preventing lung injury during viral infections. Therefore, the jury is out regarding the role of ACE2 levels in SARS-CoV2 infections, and its role may differ between the upper and lower respiratory tract.

The serious damage caused by coronaviruses such as SARS-CoV1 and 2 is due to their infection of the lower airways with rapid virus replication, massive inflammatory cell infiltration producing a huge increase in proinflammatory cytokines and chemokines leading to acute respiratory distress syndrome (ARDS), the major cause of morbidity and mortality [12]. The initial infection of the airway epithelium leads to rapid viral replication [13, 14] complicated by a virus induced delayed increase in class 1 interferon (IFN*α*/β) expression in dendritic cells (DC) that would normally block viral replication and enhance viral clearance by CD8 T cells [15]. The delayed expression of IFN*α*/β subsequently increases recruitment of proinflammatory cells, contributing to the problem. These infected airway epithelial cells then secrete a number of proinflammatory cytokines/chemokines that further dysregulate the innate immune response and attract the influx of inflammatory cells including neutrophils, monocytes and macrophages while sensitizing T cells to apoptosis [16]. The consequences include a breakdown in the microvascular and alveolar epithelial barrier from apoptosis of the lung epithelium and endothelium resulting in vascular leakage and alveolar edema. The T cell response required for viral clearance is blunted [17], and their role in dampening the cytokine storm is reduced. However, recent studies evaluating cells from bronchopulmonary lavage (BAL) of patients infected with SARS-CoV2 vs other organisms using single cell RNA-seq to define the different cell populations and what they expressed observed that in severe SARS-CoV2 pneumonia there was a high number of T cells thought to be recruited by infected macrophages expressing cytokines and chemokines such as CCL4 and CXCL10 that in turn expressed proinflammatory cytokines such as interferon-gamma (IFNγ) that further stimulated the inflammatory macrophages [18, 19]. Moreover, SARS-CoV2 infection increases the numbers of myeloid derived suppressor cells in the lungs that could contribute to suppression of the immune response to the initial infection allowing the infection to spread [20]. So where does vitamin D fit in?

## Vitamin D and viral respiratory tract infections

Vitamin D insufficiency has been linked to increased risk of respiratory tract infections including SARS-CoV2 in a large number of association studies [21–29], whereas vitamin D supplementation appears to be protective especially in those with low levels of 25OHD at baseline and treated with modest doses at daily or weekly intervals [28, 30, 31]. Similarly, certain vitamin D receptor (VDR) polymorphisms have been associated with increased risk of acute lower respiratory tract infections [32]. However, the results from randomized clinical trials (RCTs) examining the ability of vitamin D supplementation to limit viral infections have been mixed, as recently reviewed [28, 33]. Vitamin D insufficiency has also been associated with a large number of other viral infections including EBV, VZV, CMV, RSV, HIV, HCV, HBV, HPV, Dengue [33, 34], but until RCTs determine whether the lower levels of 25OHD (marker of vitamin D insufficiency) are causal vs consequential (reverse causality), the role of vitamin D in these diseases remains uncertain. To this point, a mendelian randomization study which attempted to circumvent the problem of reverse causality by evaluating whether single nucleotide polymorphisms (SNPs) associated with 25OHD levels in genome wide association studies (GWAS) differed between patients infected with SARS-CoV2 and the general population did not find a correlation between those SNPs associated with 25OHD levels and infection rates [35].

## Pulmonary immune defense mechanisms and their regulation by vitamin D signaling

The respiratory tract has a large surface area (approximately 70m^2^) in contact with the environment. Thus it provides a major site for invasion by pathogenic organisms, against which it must defend. The defense mechanism is comprised of both innate and adaptive immunity. Activation of the innate immune system drives activation of the long term adaptive immune system [36]. The principal cells involved are the airway epithelia, alveolar macrophages, and dendritic cells (DC). All of these cells express CYP27B1, the enzyme producing 1,25(OH)_2_D, the active metabolite of vitamin D, as well as the vitamin D receptor (VDR) [37–39]. Expression of CYP27B1 is constitutive in airway epithelial cells [37], although it can be further increased by some types of viral infection [40]. The 1,25(OH)_2_D produced by these cells promotes alveolar epithelial cell proliferation and reduces their apoptosis after an inflammatory challenge [41]. Deletion of VDR from these cells leads to loss of integrity of the epithelium [42]. On the other hand CYP27B1 is induced in alveolar macrophages by toll like receptor (TLR) ligands for TLR1/2 (such as the lipopeptide from *mycobacterium tuberculosis*), interferonγ (IFNγ), and LPS [43, 44], and in DC by TNFα, IFNγ, polyI:C, and LPS [45–47]. Moreover, these cells all express pattern recognition receptors (PRRs) of which TLRs are a major component and by which viral RNAs are recognized [48].

### Innate immune response

The innate immune response is initiated with the activation of PRRs in the cells of the respiratory tract. There are 10 functional TLRs in human cells (of 11 known mammalian TLRs). TLRs are an extended family of host noncatalytic transmembrane PRRs that interact with specific membrane patterns (PAMP) shed by infectious agents such as viruses that trigger the innate immune response in the host. A number of these TLRs signal through adapter molecules such as myeloid differentiation factor-88 (MyD88) and the TIR-domain containing adapter inducing IFN-β (TRIF). MyD88 signaling includes translocation of NFĸB to the nucleus, leading to the production and secretion of a number of inflammatory cytokines. Indeed one of the anti-inflammatory actions of 1,25(OH)_2_D is to block NFĸB translocation to the nucleus where it would otherwise induce a panel of proinflammatory cytokines. This is achieved in part by inducing the inhibitor for NFĸB translocation, IĸBα, that binds NFĸB and keeps it in the cytoplasm [49]. This effect of 1,25(OH)_2_D does not deter its influence on viral clearance [50]. TRIF signaling leads to the activation of interferon regulatory factor-3 (IRF-3) and the induction of type 1 interferons such as IFNα/β. MyD88 mediates signaling from TLRs 2, 4, 5, 7 and 9, whereas TRIF mediates signaling from TLR 3 and 4. TLR1/2, TLR4, TLR5, TLR2/6 respond to bacterial ligands, whereas TLR3, TLR7, and TLR 8 respond to viral ligands. CD14 serves as a coreceptor for a number of these TLRs. CD14 is induced by 1,25(OH)_2_D [37]. Activation of TLRs leads to the induction of antimicrobial peptides (AMPs) and reactive oxygen species (ROS), which kill the organism. However, excess ROS can also cause cell damage. 1,25(OH)_2_D can control this process by inducing antioxidant genes such as glutathione synthase [51]. Among the antimicrobial peptides produced by the activated cells is cathelicidin. Cathelicidin plays a number of roles in the innate immune response. The precursor protein, hCAP18, must be cleaved to its major peptide LL-37 to be active. In addition to its antimicrobial properties, LL-37 can stimulate the release of cytokines such as IL-6 and IL-10 through G protein coupled receptors, and IL-18 through ERK/P38 pathways, stimulate the EGF receptor leading to activation of STAT1 and 3, modulate TLR signaling including enhanced recognition of dsRNA, increase binding of dsRNA binding to cell surface scavenger receptors increasing endocytosis, induce the chemotaxis of neutrophils, monocytes, macrophages, and T cells into the site of infection, and promote the clearance of respiratory pathogens by inducing apoptosis and autophagy of infected epithelial cells [34, 52]. The expression of this antimicrobial peptide is induced by 1,25(OH)_2_D in both myeloid and epithelial cells [53–55]. 1,25(OH)_2_D also induces another AMP, defensin β2, albeit somewhat indirectly as it involves induction of NOD2/card15/IBD1, a pattern recognition receptor for muramyl dipeptide that in turn activates NOD2 to stimulate NFĸB, the direct inducer of defensin β2 [56]. Defensin β2, like cathelicidin, contributes to host defense by stimulating the expression of antiviral cytokines such as IFNβ, immune inducing molecules (NOD2, TNFα, IL-1β, IL-6), and chemokines involved in the recruitment of monocytes/macrophages, natural killer cells, neutrophils, T cells and DC [57]. In summary the innate immune system is the first line of defense against invading pathogens initiating the inflammatory response and activating the adaptive arm of the immune defense mechanism [58]. However, chronic activation of the innate immune response can be deleterious. 1,25(OH)_2_D inhibits TLR2, 4, 9 expression in monocytes in the later stages of activation [59, 60] and limits the excessive production of TNFα and IL-12 [61] serving to modulate chronic innate immune activity. But an additional part of the cytokine storm is due to an uncontrolled adaptive immune system, which vitamin D signaling is also poised to modulate.

### Adaptive immune response

The adaptive immune response is initiated by cells specialized in antigen presentation, DC and macrophages in particular, activating the cells responsible for subsequent antigen recognition, T and B lymphocytes. These cells are capable of a wide repertoire of responses that ultimately determine the nature and duration of the immune response. Activation of T and B cells occurs after a priming period in tissues of the body, eg. lymph nodes, distant from the site of the initial exposure to the antigenic substance, and is marked by proliferation of the activated T and B cells accompanied by post translational modifications of immunoglobulin production that enable the cellular response to adapt specifically to the antigen presented. I will not focus on the role of B cell activation and immunoglobulin production, but limit this discussion to the T cells. The type of T cell activated, CD4 or CD8, or within the helper T cell class Th1, Th2, Th17, Treg, and subtle variations of those, is dependent on the context of the antigen presented by which cell and in what environment. Systemic factors such as vitamin D influence this process. 1,25(OH)_2_D in general exerts an inhibitory action on the adaptive immune system. When airway DC are activated as by a virus, they migrate to lymph nodes where they gain enhanced ability to present antigen for activation of T cells [62]. As noted previously this activation and maturation of DC includes increased CYP27B1 expression but also a decrease in VDR [46]. 1,25(OH)_2_D decreases the maturation of DC as marked by inhibited expression of the costimulatory molecules HLA-DR, CD40, CD80, and CD86, decreasing their ability to present antigen and so activate T cells [63]. Furthermore, by suppressing IL-12 production, important for Th1 development, and IL-23 and IL-6 production important for Th17 development and function, 1,25(OH)_2_D inhibits the development of Th1 cells capable of producing IFNγ and IL-2, and Th17 cells producing IL-17 [64]. These actions prevent further antigen presentation to and recruitment of T lymphocytes (role of IFNγ), and T lymphocyte proliferation (role of IL-2). Furthermore, suppression of IL-12 increases the development of Th2 cells leading to increased IL-4, IL-5, and IL-13 production, which further suppress Th1 development shifting the balance to a Th2 cell dominated profile. Treatment of DCs with 1,25(OH)_2_D can also induce CD4 + /CD25 + regulatory T cells (Treg) cells [65] as shown by increased FoxP3 expression, critical for Treg development [64]. These cells produce IL-10, which suppresses the development of the other Th subclasses. Treg are critical for the induction of immune tolerance [66] and likely play a key role in preventing the cytokine storm associated with severe respiratory disease caused by viral infections such as SARS-CoV2 [67]. On the other hand myeloid suppressor cells, that are increased in SARS-CoV1 infections, may suppress the initial immune response to the infection, facilitating its spread. These cells like other immune cells express VDR, and 1,25(OH)_2_D blocks their differentiation and so their suppressive action [20].

1,25(OH)_2_D has both direct and indirect effects on regulation of a number of key cytokines involved with the proinflammatory response. TNFα has a vitamin D response element (VDRE) in its promoter to which the VDR/RXR complex binds, leading to its repression. As noted earlier 1,25(OH)_2_D both blocks the activation of NFĸB via an increase in IĸBα expression and impedes its binding to its response elements in the proinflammatory genes such as IL-8 and IL-12 that it regulates. 1,25(OH)_2_D has also been shown to bring an inhibitor complex containing histone deacetylase 3 (HDAC3) to the promoter of rel B, one of the members of the NFĸB family, suppressing gene expression. Thus, TNF/NFkB activity is markedly impaired by 1,25(OH)_2_D at multiple levels. Furthermore, 1,25(OH)_2_D suppresses IFNγ, and a negative VDRE has been found in the IFNγ promoter. GM-CSF is regulated by VDR monomers binding to a repressive complex in the promoter of this gene, competing with nuclear factor of T cells 1(NFAT1) for binding to the promoter. As implied in the introduction, we do not know how much of the knowledge gained about vitamin D regulated immunity in other systems applies to SARS-CoV2 infections given its recent appearance on the scene, but these studies do underlie the hope that vitamin D will be of some help in the prevention/treatment of this pandemic given how few other interventions with the exception of vaccination and perhaps newly developed oral antivirals are proving useful.

## Conclusion

While the data clearly demonstrating a beneficial role for vitamin D in SARS-CoV2 infections are limited, its role in regulating both the innate and adaptive immune systems certainly suggests that it may be. The innate immune system is the first line of defense against invading pathogens such as viruses. It is prebuilt, relying on constitutive expression of pattern recognition receptors like TLRs to identify such pathogens. 1,25(OH)_2_D enhances that defense by inducing AMPs like cathelicidin that lead to viral destruction and clearance by several mechanisms, help recruit neutrophils, monocytes/macrophages, and DC which further the killing and clearance of these pathogens, and initiate the adaptive immune response. While beneficial acutely, chronic activation of the innate immune response is not, and can result in the cytokine storm. 1,25(OH)_2_D works to curtail this chronic innate immune response through a number of mechanisms including down regulation of TLRs and direct inhibition of TNF/NFĸB and IFNγ signaling pathways. The adaptive immune system provides a more specific response, but takes longer to develop, although once developed provides a powerful response against invading organisms. However, this response if not controlled can also be destructive. Vitamin D, via its active metabolite 1,25(OH)_2_D, regulates adaptive immunity by limiting the maturation of DC, limiting their ability to present antigen to T cells, and shifting the T cell profile from the proinflammatory Th1 and Th17 subsets to Th4 and Treg subsets, which inhibit the proinflammatory processes. Although these results come from studies with a variety of pathogens, viral and bacterial, the relevance of these protective actions on SARS-CoV2 merits further investigation.
